# Extended Stability of Ascorbic Acid in Pediatric TPN Admixtures: The Role of Storage Temperature and Emulsion Integrity

**DOI:** 10.3390/pharmaceutics17111375

**Published:** 2025-10-24

**Authors:** Rafał Chiczewski, Żaneta Sobol, Alicja Pacholska, Dorota Wątróbska-Świetlikowska

**Affiliations:** Department of Pharmaceutical Technology, Pomeranian Medical University in Szczecin, Rybacka 1, 70-204 Szczecin, Polanddorota.watrobska.swietlikowska@pum.edu.pl (D.W.-Ś.)

**Keywords:** parenteral nutrition, pediatric TPN, vitamin C, ascorbic acid stability, emulsion integrity, storage temperature

## Abstract

**Background/Objectives:** This study assessed the chemical and physical stability of ascorbic acid in pediatric total parenteral nutrition (TPN) admixtures under conditions reflecting both hospital compounding and home administration. **Methods:** Two storage protocols were examined: (A) refrigerated storage (15 days, 4 ± 2 °C) followed by addition of ascorbic acid and a 24-h period of storage at room temperature, and (B) vitamin supplementation within 24 h after composing and storage at 21 ± 2 °C. A validated high-performance liquid chromatography (HPLC) method was used to quantify ascorbic acid degradation. Physical stability was evaluated via optical microscopy, dynamic light scattering (DLS), laser diffraction (LD), zeta potential, and pH measurement. **Results:** Ascorbic acid content remained above 90% of the declared value in both protocols, although gradual degradation was observed with increasing storage time and temperature. Emulsion droplet sizes remained within pharmacopeial limits (<500 nm), and no coalescence or phase separation was detected. Zeta potential values (−20 to −40 mV) confirmed kinetic stability, while pH ranged from 5.8 to 6.2, remaining within acceptable safety margins. **Conclusions:** Vitamin C in pediatric TPN admixtures is stable under refrigerated conditions for up to 15 days. However, the additional 24 h at room temperature resulted in measurable loss of ascorbic acid content, suggesting a need for improved guidance in home-based parenteral nutrition, particularly regarding transport and handling. The study underscores the importance of strict cold-chain maintenance and highlights the role of emulsion matrix and packaging in protecting labile vitamins. This research provides practical implications for hospital pharmacists and caregivers, supporting better formulation practices and patient safety in pediatric home TPN programs.

## 1. Introduction

Parenteral nutrition (PN) entails the direct administration of balanced and essential nutrients into the bloodstream, which is crucial for the proper development and functioning of the body. This method is employed for patients who are unable to tolerate oral or enteral feeding. It is considered a last resort after other forms of feeding have been conclusively excluded. Over the years, PN has evolved into an indispensable life-sustaining therapy in various clinical contexts, utilized both in hospitalized patients and in home care for chronically ill individuals. Prior to initiating total parenteral nutrition (TPN) therapy, it is imperative to ascertain the patient’s nutrient requirements, given the 100% bioavailability following intravenous administration. This ensures that the entire administered dose is absorbed by the body. For PN to be effective, three key conditions must be met [1,2]:

1. The nutrient amounts required to sustain life must fulfill the patient’s metabolic and energetic needs.
2. The ratio between the nitrogen content and the energy provided by non-protein components (where 1 g of nitrogen should equate to 130–200 kcal) must be appropriate.
3. The ratio between carbohydrates and lipids (50–75 kcal from carbohydrates for every 25–50 kcal from lipids) must be balanced to ensure the proper utilization of these components in the body’s biochemical processes.

TPN mixtures are large volume, sterile infusions prepared under aseptic conditions, administered via a catheter directly into the central or peripheral vein. The individual components are added to a sterile bag, creating a comprehensive pharmaceutical form of the drug as a single, easy-to-use infusion system, known as “All in one” (AIO). Another form is the “Two in one” system (TIO), wherein one compartment contains glucose and amino acids with added electrolytes, and the other contains a submicron emulsion with vitamins. This configuration permits the observation of the solution’s clarity throughout the storage and administration period to the patient [3].

There is a significant difference in durability (“shelf life”) between the “All in One” and “Two in One” systems. The composition of AIO is adapted to the patient’s therapeutic requirements and provides individualized therapy. A large number of components in a single compartment increases the critical aggregation number (CAN). There is a strong connection between high polyvalent ion usage and increased CAN levels. Exceeding the calculated electrolyte concentration may lead to coalescence, as a result of which lipid droplets combine into larger aggregates. This is an irreversible stage that enlarges the size of the lipid droplets. Phase separation in the emulsion prevents the safe administration of PN to the patient. Despite the clinical and economic benefits of AIO systems, their disadvantage is the difficulty in achieving satisfactory durability and compatibility of a given composition. The shelf life of complete mixtures for parenteral nutrition, prepared in single compartment bags are limited and is usually up to 48 h [4,5,6,7]. Separation of the lipid phase from the glucose and amino acids extends durability. Preliminary PN is prepared in two-chamber bags, which are combined immediately before administration to the patient. The durability of preliminary mixtures is limited and should be determined based on physicochemical analysis individually for each composition. Available ready-made pre-mixtures are stable for up to 18 months before bag activation. After mixing the chambers as directed it can be stored for up to 7 days at room temperature or up to 14 days when refrigerated (including the time of administration) in exceptional situations [8,9].

Hospital pharmacists encounter numerous challenges in incorporating vitamins and trace elements into Total Parenteral Nutrition (TPN) mixtures. Various authors recommend against adding vitamins and trace elements to the same mixture due to potential incompatibilities and instabilities. It is well-established that ascorbic acid (AA) is the least stable water-soluble vitamin, primarily because of its oxidizing properties, a reaction catalyzed by divalent ions. Given the insufficient evidence regarding the compatibility of trace elements with vitamins, some researchers propose administering vitamins and trace elements via two separate but concurrent intravenous infusions. The supplementation of neonates is further complicated by the necessity for continuous administration of all components over extended periods [10]. In numerous hospitals, vitamins and trace elements are added to the PN mixture immediately before administration. Conversely, vitamins serve as a protective role against lipid peroxidation in emulsions, suggesting that vitamins should be included with other excipients in parenteral mixtures. The stability of PN mixtures encompasses both physical and chemical stability. A critical parameter is the size of lipid emulsion droplets, which must be smaller than the smallest blood vessels to minimize the risk of embolism, a significant patient safety concern. The evaluation of droplet size and distribution, along with electrophoretic mobility (zeta potential), is essential for ensuring the kinetic stability of the lipid emulsion [11].

Ascorbic acid plays an essential biochemical role in the body, making it a crucial component of parenteral nutrition (PN). It is a potent antioxidant and an enzyme cofactor. The determination of daily intravenous requirements is based on plasma concentrations, which fluctuate during inflammatory responses. The lack of a definitive deficiency marker means that an ascorbate concentration in serum or plasma below 0.3 mg/dL indicates an inadequate level of vitamin C [12]. Pharmacokinetic data suggest that the appropriate dose for pediatric patients receiving PN is 100 mg per day, while for adult patients, it is 200 mg per day. Ascorbic acid is the least stable vitamin added to PN, rapidly oxidizing, especially at high temperatures and in the presence of copper ions. Ascorbic acid (AA) converts into dehydroascorbic acid (DHAA), which also exhibits biological activity. Subsequent transformation stages are irreversible, and the intermediate products do not possess biological activity. Vitamin C readily oxidizes in solutions with a pH above 4. The addition of vitamin C and other trace elements to PN mixtures can lead to precipitation or degradation of other nutrients. Oxygen, the primary factor responsible for the degradation of vitamins in PN mixtures, originates from the filling process or oxygen permeation through the bag walls [4,11,13].

The stability of PN is affected by storage temperature and duration. During storage, lipid hydrolysis occurs, leading to the release of free fatty acids, which results in a pH decrease. A reduced pH accelerates the degradation of vitamins. Elevated temperatures increase the risk of lipid coalescence. The intensity of physicochemical changes in PN is directly proportional to the time of storage [9,14].

The objective of this study was to quantify ascorbic acid in pediatric total parenteral nutrition admixtures intended for home parenteral nutrition, examining the impact of time on vitamin C degradation in these admixtures. Previous studies have evaluated vitamin C stability in commercial TPN admixtures over short periods (up to 7 days), but limited data exist on extended storage periods under refrigerated conditions followed by room temperature exposure, particularly in home PN settings. Additionally, the study aimed to evaluate the physical stability of the admixtures by characterizing droplet size distribution, average droplet size, zeta potential, and pH measurements. Clinical TPN lipid formulations are designed as oil-in-water (O/W) emulsions stabilized by surfactants; this architecture underpins the droplet-size targets verified by LD/DLS and microscopy in the present study.

## 2. Materials and Methods

### 2.1. Preparation of Parenteral Nutrition Admixtures

This experimental study aimed to evaluate the chemical and physical stability of pediatric parenteral nutrition (PN) admixtures, with a particular focus on ascorbic acid (vitamin C) degradation under varying storage conditions and time points. The lipid emulsion used throughout is an oil-in-water (O/W) system, i.e., oil droplets dispersed in an aqueous continuous phase. Two vitamin addition strategies were employed to mimic hospital versus home-based PN practices. Group A samples were stored refrigerated (4 ± 2 °C) for 15 days prior to vitamin supplementation and then stored an additional 24 h at room temperature, while Group B samples received vitamin supplementation 24 h post-compounding and were subsequently stored at room temperature (21 ± 2 °C) for 24 h. The impact of these variables on vitamin C content and emulsion integrity was assessed.

PN admixtures were aseptically prepared under controlled environmental conditions by hospital pharmacists. Formulations were based on clinical standards and reflect compositions used at the *Children’s Memorial Health Institute in Warsaw,* (Poland). Each admixture included

- Carbohydrate source: Glucose 50% solution.
- Protein source: Aminoven Infant^®^.
- Lipid emulsion: Smoflipid^®^ or an equivalent product.
- Electrolytes: Glycophos^®^, 10% NaCl, 15% KCl, 20% MgSO_4_, and calcium gluconate. Ascorbic acid: Soluvit^®^ N, Cernevit^®^ are multivitamin products (1 mL of Soluvit^®^ N is equivalent to 12 mg of Vitamin C and 1 mL of Cernevit^®^ is equivalent to 10 mg of Vitamin C).
- Trace elements: Peditrace^®.^
- Diluent: Water for injection (*WFI*).

**Figure 1:**
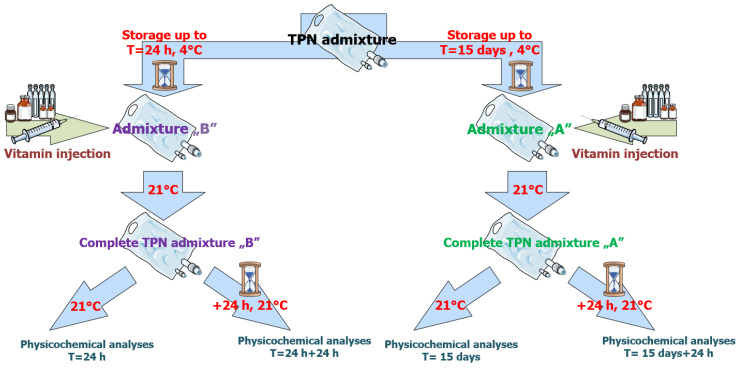
Scheme of TPN admixture preparation and physiochemical analyses.

Vitamin supplementation was performed in two formats. For Group B, vitamins were added 24 h after compounding, simulating typical hospital pharmacy workflow. For Group A, vitamins were added after 15 days of refrigerated storage to simulate patient addition at home. Water-soluble vitamins (Soluvit^®^ N) were reconstituted in Vitalipid^®^ Infant, while Cernevit^®^ was reconstituted in 0.9% NaCl. The solutions were gently agitated until fully dissolved and passed through a 0.22 μm syringe filter before incorporation into the PN bag. All PN bags were gently mixed following supplementation (Figure 1). A detailed composition of these PN admixtures is presented in Table 1.

### 2.2. Storage and Sampling Protocol

Prepared PN admixtures were filled into one-compartment ethylene vinyl acetate (EVA) bags, which offer superior flexibility and oxygen barrier properties. Bags were labeled, and stored under assigned conditions:

- Group A: 15 days of storage at 4 ± 2 °C, followed by vitamin addition and additional 24 h of storage at 21 ± 2 °C with light protection (t = 15 days, and t = 15 days + 24 h).
- Group B: Vitamins added at 24 h post-preparation, followed by an additional 24 h of storage at 21 ± 2 °C (t = 24 h, and t = 24 h + 24 h).

Prior to sampling, the bags were conditioned at room temperature for 2 h to simulate patient preparation time. Samples were collected using sterile syringes and mixed thoroughly to ensure uniformity. The scheme of PN admixtures preparation process and physiochemical analyses in Figure 1.

### 2.3. Visual Inspection and Organoleptic Evaluation

Visual inspection was conducted by two independent observers trained in PN admixture assessment. Bags were evaluated against black and white backgrounds under standardized lighting conditions. Signs of physical instability—such as creaming, flocculation, or phase separation—were recorded. Particular attention was paid to changes in opacity, color shift, or formation of visible lipid layers.

### 2.4. Optical Microscopy for Lipid Droplet Analysis

10 mL aliquots from each admixture were placed on microscopy slides and analyzed under a digital optical microscope equipped with a calibrated ocular micrometer (*Nikon Microscope Eclipse Ei* (Amstelveen, The Netherlands) with 0.01 mm precision). Fifteen visual fields (five per sample, triplicate preparations) were inspected at 40× magnification. Droplets > 5 μm in diameter were counted, and the largest diameter per field was recorded. Samples were equilibrated at 21 ± 2 °C before imaging. Images were captured at ≥300 dpi and exported with embedded scale bars (µm). Photographic documentation was obtained for archival and comparative analysis.

### 2.5. Particle Size Distribution Analysis

Particle size distribution and polydispersity were measured at 21 ± 2 °C via three complementary techniques:

1. Dynamic Light Scattering (DLS): Samples were diluted 1:100 with water for injection (WFI) and measured using a Zetasizer Nano ZS (Malvern Panalytical Ltd., Malvern, UK). Z-average size and polydispersity index (PDI) were recorded. The PDI threshold for acceptable stability was <0.2.
2. Laser Diffraction (LD): Droplet volume distribution was determined using a Mastersizer 3000 (Malvern Panalytical Ltd., Malvern, UK). Approximately 2 mL of admixture was diluted into 500 mL of WFI under stirring. D_10_, D_50_, and D_90_ values were calculated, reflecting the droplet diameter at which 10%, 50%, and 90% of the total volume is composed of smaller particles.
3. Optical Microscopy: As described above, used as a qualitative check.

### 2.6. Zeta Potential Measurement

Zeta potential was assessed using the Zetasizer Nano ZS by electrophoretic light scattering. Samples were diluted 1:500 with WFI and transferred to single-use folded capillary cells. Three independent readings per sample were obtained. Zeta potential values more negative than −20 mV were interpreted as indicative of good kinetic stability and low aggregation propensity.

### 2.7. pH Measurement

10 mL of parenteral admixtures were used for each measurement. The pH measurements were made at room temperature at 21 ± 2 °C by direct immersion of the electrode in the admixture. pH was measured using a pH/Ion meter (IKA, Darmstadt, Germany). The meter was calibrated daily using pH 4.0 and 7.0 standard buffers. All measurements were performed in triplicate at room temperature, and the electrode was rinsed with WFI between samples.

### 2.8. Quantification of Ascorbic Acid via HPLC

Vitamin C content was determined using a validated high-performance liquid chromatography (HPLC) method. Admixture samples were mixed 1:1 with a stabilizing reagent (3.5% phosphorous acid + 1 mM EDTA) to prevent oxidative degradation. Samples were centrifuged by Centrifuge Optima L100-XP, Beckman Coulter, Indianapolis, IN, USA, (15,000 rpm, 15 min) and filtered through a 0.22 µm PVDF membrane.

Chromatographic separation was carried out using a reverse-phase C_18_ column (Kinetex^®^ 5 μm C18 Phenomenex, Torrance, CA, USA, 250 mm × 4.6 mm, 5 µm) at 25 °C. The mobile phase consisted of 0.05 M KH_2_PO_4_ buffer (pH 3.0), filtered and degassed before use. Detection was performed at 254 nm using a diode-array detector (DAD—MD-4010 JASCO, Tokyo, Japan). The flow rate was 0.4 mL/min (pump—PU-4180 JASCO, Japan), and 10 µL of sample was injected for each run [15,16].

### 2.9. Statistical Analysis

All experimental data were processed using Statistica 13 (StatSoft, Kraków, Poland). Results are presented as mean ± standard deviation (SD). Differences between groups and time points were analyzed using the Friedman test for repeated measures. Pairwise comparisons were made using the Mann–Whitney U test. A *p*-value < 0.05 was considered statistically significant.

## 3. Results and Discussion

### 3.1. Microscopic Observations

**Figure 2:**
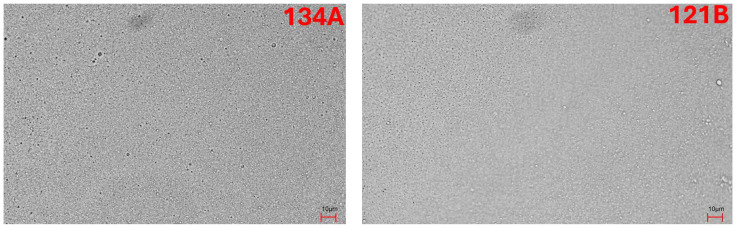
Representative photomicrograph of TPN 134A after t = 24 h + 24 h of storage, TPN 121B, after t = 15 days of storage and TPN 141a, after t = 15 days + 24 h of storage.

Microscopic evaluation of the emulsions revealed that lipid droplet size remained within acceptable limits throughout the observation period. None of the samples displayed lipid globules exceeding 5 µm, which is a critical safety threshold defined in pharmacopeial standards to mitigate the risk of capillary blockage. The majority of droplets measured ranged between 200 µm and 400 µm, confirming the uniformity and stability of the emulsions under the tested storage conditions. Under microscopic observation, no oil droplets larger than 2 μm were detected in the parenteral admixtures (Figure 2) suggesting that the parenteral admixtures were stable and could be considered as safe for patients when administered intravenously. No phase separation or coalescence was observed. Optical microscopy allowed for determination of the higher diameters of lipid globules (Figure 2).

### 3.2. Dynamic Light Scattering (DLS) and Laser Diffraction (LD)

Table 2 presents the evolution of lipid droplet size over time, determined using the laser diffraction (LD) method. Parameters measured included Dx_(10)_, Dx_(50)_, and Dx_(90)_, corresponding to the diameters below which 10%, 50%, and 90% of the lipid droplets are found. Measurements were conducted at four distinct time points: after 24 h, 24 h plus an additional 24-h incubation period, 15 days, and 15 days plus 24 h of storage. Both sample groups, denoted as “A” and “B,” were assessed under identical conditions. Throughout the storage period, the median droplet diameter (Dx_(50)_) remained within the range of approximately 190–295 µm, while 90% of the lipid droplets (Dx_(90)_) consistently remained below 520 µm. These findings are in agreement with pharmacopeial requirements for lipid injectable emulsions, which mandate that no more than 90% of lipid globules exceed 500 µm in diameter. No droplet sizes greater than 1 µm were detected in any of the admixtures, confirming the absence of coalescence and phase separation [17]. Statistical analysis indicated no significant differences (*p* < 0.05) between samples A and B in terms of Dx(90) values at early time points (24 h and 15 days). However, a statistically significant difference was observed at 15 days + 24 h (*p* = 0.00024), with samples B exhibiting slightly higher Dx_(90)_ values. This may suggest early signs of emulsion destabilization upon prolonged storage in certain conditions, although all values remained within acceptable pharmacopeial limits. These results confirm the overall physical stability of the lipid emulsions over time. The LD method proved effective in detecting subtle shifts in the upper range of droplet size distribution, providing valuable insight into the potential onset of instability.

### 3.3. Zeta Potential

Table 2 presents the physicochemical stability of total parenteral nutrition (TPN) admixtures was assessed using dynamic light scattering (DLS) by evaluating the Z-average particle size, polydispersity index (PDI), zeta potential, and electrical conductivity at four storage intervals: 24 h, 24 h + 24 h, 15 days, and 15 days + 24 h. Measurements were performed for two sets of admixtures, labeled as samples A and samples B.

**Figure 3:**
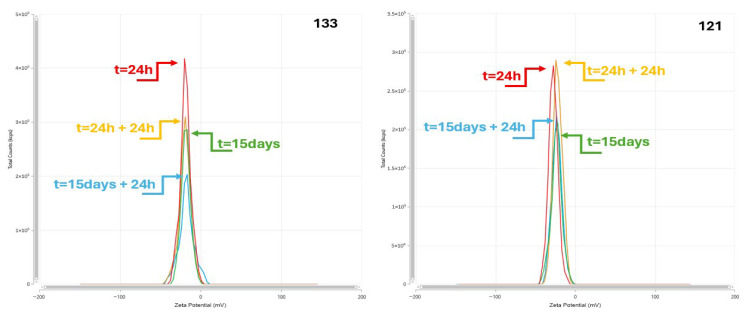
Size distribution of oil phase droplets in the TPN 121 and TPN 133 admixtures by the PCS method.

The Z-average diameters of lipid droplets ranged from approximately 210 to 270 nm across all samples, with most values concentrated between 230–250 nm, indicating a stable nanoemulsion. The observed size distribution aligns with USP <729> requirements, where the mean droplet diameter obtained by light scattering must not exceed 500 nm for injectable lipid emulsions (Figure 3).

The PDI ranged from 0.03 to 0.27, with the majority of values <0.20, indicating a relatively narrow droplet-size distribution in line with prior emulsion reports using DLS. The zeta potential of the emulsions remained consistently negative, ranging from −12.7 mV to −39.7 mV [18,19]. These values indicate electrostatic repulsion between droplets, which contributes to the colloidal stability of the emulsions. Over the storage period, a progressive increase in the negative charge was noted, especially between day 0 and day 15, suggesting increasing emulsion stability due to stronger repulsive forces. This trend was observed in both types of samples.

Conductivity measurements remained within a relatively low range (~0.015–0.2 mS/cm), consistent with the expected ionic content of TPN emulsions. No sharp increases were noted, indicating that no significant degradation or phase separation occurred over time. No statistically significant differences (*p* > 0.05) were detected in Z-average, PDI, or zeta potential between samples A and B at any time point, confirming the comparable stability of both formulations throughout storage. Minor numerical fluctuations were attributed to inherent batch variability or measurement conditions but did not translate into functional instability (Table 3).

### 3.4. pH Measurement

The pH values of all admixtures ranged from 5.8 to 6.2, with minor fluctuations that were not statistically significant (*p* > 0.05). These values are within the acceptable range for parenteral administration and are conducive to maintaining the chemical integrity of ascorbic acid. A slight decline in pH was observed after 24 h of exposure to room temperature, which is consistent with lipid hydrolysis and mild acidification over time. However, the changes were not sufficient to compromise the physical or chemical stability of the admixtures. If the pH of a parenteral admixture is noted to be lower than 5.0, it could be unstable and unsafe for the patient [6,20]. The pH range for the commercial lipid emulsions used for parenteral admixtures is between 6.0–9.0. This pH maintains the negative charge of the oil globules and guarantees the stability of the lipid emulsion (Table 4).

### 3.5. Ascorbic Acid Stability (HPLC Results)

Quantitative determination of ascorbic acid levels was performed using a validated HPLC method. The method was shown to be specific, linear, precise and accurate for the quantitative analysis of ascorbic acid in parenteral nutrition admixtures. Specificity was confirmed by comparing chromatograms of standard vitamin solutions with chromatograms of TPN admixtures without added vitamin (placebo). The purity of each chromatographic peak was assessed using diode-array detection to confirm the presence of single-component peaks without interference from other matrix constituents.

Linearity was evaluated across five concentration levels in the range of 10–200 µg/mL. Method precision was assessed both intra-day and inter-day by preparing samples at 80%, 100%, and 120% of nominal concentration. Accuracy was verified by recovery testing of vitamin C in spiked parenteral placebo solutions. Calibration was performed with AA standards (10–200 µg/mL; R^2^ > 0.999). Limit of detection (LOD) and quantification (LOQ) were 0.3 µg/mL and 1.0 µg/mL, respectively. The linearity of the method was determined by linear regression analysis of the values obtained experimentally with the software Excel (*Microsoft Excel 16.0 2024*, Redmond, WA, USA).

As shown in Table 5, the ascorbic acid content remained stable over the initial 24-h storage period at 4 ± 2 °C, with mean recovery rates above 96% of the theoretical value (96.01% ± 0.03). No statistically significant degradation (*p* > 0.05) was observed between 24 and 48 h, as evidenced by identical mean concentrations at both time points (96.01% ± 0.03). After 15 days of cold storage, a modest decrease in ascorbic acid concentration was observed. with average content declining to 94.07% ± 0.06. Further exposure to ambient temperature and light for an additional 24 h led to a continued decline to 93.11% ± 0.05. Despite the progressive reduction, all measured concentrations remained above 90% of the declared value, thus complying with pharmacopeial criteria for acceptable stability in parenteral preparations. These results are consistent with previously published studies indicating ascorbic acid is sensitivity to temperature and light, particularly beyond the standard 24-h infusion period. Importantly, no critical threshold was exceeded that would compromise the clinical utility or safety of the TPN admixtures. The data confirm that ascorbic acid retains adequate chemical stability under refrigerated conditions for up to 15 days and that short-term thermal exposure does not lead to excessive degradation. The lipid emulsion matrix likely contributed to light protection, as suggested in earlier literature.

Previous investigations into the stability of TPN admixtures have primarily focused on short-term hospital storage conditions. Turmezei et al. [21] evaluated the physicochemical stability of infant TPN admixtures at 2–8 °C, 25 °C, and 30 °C over a 14-day period, assessing particle size, zeta potential, and the degradation of ascorbic acid and L-alanyl-L-glutamine. While their study provided valuable insights into temperature-dependent changes, it presented several methodological limitations: the analytical approach (ESI-MS/MS) required sample ultrafiltration and was not applicable in routine hospital pharmacy settings, and the degradation of vitamin C was assessed only within the first 48 h without considering real-world handling and transport conditions. In contrast, the present study extends these observations by investigating the long-term stability of ascorbic acid in pediatric TPN admixtures designed for home parenteral nutrition, including a 15-day refrigerated storage period followed by 24 h at room temperature to simulate patient use. The use of a validated HPLC-DAD method enabled direct quantification of ascorbic acid without sample pretreatment, providing a practical and reproducible analytical tool suitable for hospital and compounding pharmacy environments. Furthermore, the inclusion of complementary physicochemical analyses—dynamic light scattering, laser diffraction, zeta potential, and pH monitoring allowed for a more comprehensive evaluation of emulsion integrity. These findings further support the use of HPLC for routine stability testing and shelf-life determination of water-soluble vitamins in parenteral nutrition regimens, particularly those administered to patients over extended durations.

## 4. Limitations

This study reflects the practice of a single centre and the formulations used at the Children’s Memorial Health Institute in Warsaw, which limits the full generalizability of the findings to different pediatric PN (parenteral nutrition) recipes employed in other hospitals and countries. The formulations, as well as the preparation and storage protocols, were selected to faithfully mirror the clinical routine of this institution, which strengthens internal validity but narrows the scope of extrapolation. Systematic comparisons of different container materials and constructions were not performed. Monolayer EVA bags were used, whereas the literature indicates that oxygen-barrier properties and UV protection can vary across bag types and modulate the degradation rate of light-sensitive vitamins. Consequently, within the present protocol, the influence of container material on the chemical and physical stability of the admixtures was assessed only indirectly. Although the HPLC method was validated (linearity, sensitivity, precision/repeatability) and supported by DAD peak-purity analysis, a full forced-degradation study separating vitamin C from its major degradation products was not presented. Thus, the “stability-indicating” character of the method was confirmed indirectly rather than by direct demonstration of baseline separation of all key degradants. The study focused on quantifying ascorbic acid; dehydroascorbic acid and downstream oxidation products of potential clinical relevance were not monitored in parallel, which limits insight into the complete stability and safety profile of vitamin C in PN. The catalytic effect of trace metal ions (especially Cu^2+^) on ascorbic-acid oxidation, known from the literature, was discussed as background, but actual concentrations of metals in the tested admixtures were not measured. The degradation of ascorbic acid in parenteral admixtures is primarily driven by oxidative and metal-catalyzed processes. Transition metals such as copper and iron, even at trace levels, can promote the oxidation of ascorbate to dehydroascorbic acid (DHAA) through Fenton-type reactions, accelerating degradation particularly at elevated temperatures and low pH. Although the EVA packaging and lipid emulsion matrix provide partial protection against oxygen ingress and light exposure, the absence of chelating agents beyond EDTA may have permitted limited redox cycling. The degradation pathway likely followed a sequential oxidation to DHAA. The absence of these data constitutes a potential confounder that could have influenced the observed degradation rate of vitamin C. Real-life conditions of home parenteral nutrition (e.g., temperature cycling during transport, variable exposure to daylight/UV) were not fully simulated; as a result, the extent of degradation in the home setting may be under- or overestimated relative to controlled laboratory conditions. The literature also suggests that the delay to first assay due to sample-handling logistics (e.g., transport to the laboratory) can itself represent an interpretive limitation.

## 5. Conclusions

This study investigated the stability of ascorbic acid (vitamin C) in pediatric total parenteral nutrition (TPN) admixtures intended for home administration. Utilizing a validated high-performance liquid chromatography (HPLC) method, vitamin C concentrations were monitored across a 15-day storage period at 4 ± 2 °C and after an additional 24 h of exposure to ambient conditions. The findings provide critical insights into the degradation kinetics of ascorbic acid and offer valuable recommendations for clinical and pharmaceutical practice. Quantitative analysis confirmed a gradual but statistically significant reduction in ascorbic acid content over the 15-day refrigeration period, with an even more pronounced decline following subsequent exposure to room temperature. The degradation pattern was consistent across multiple TPN batches, highlighting the sensitivity of vitamin C to both time and temperature. Despite these changes, the majority of samples retained more than 90% of the initial concentration until day 15, suggesting adequate stability under standard storage conditions, although a trend toward cumulative degradation was evident. Compared to studies where lipid emulsions were present, our results suggest a slightly higher degradation rate, potentially due to reduced photoprotection. This underscores the critical role of packaging, UV shielding, and the composition of admixtures in determining overall vitamin stability. From a pharmaceutical and clinical standpoint, the preservation of ascorbic acid integrity is vital for maintaining the efficacy of TPN formulations. Ascorbic acid, being a water-soluble vitamin with potent antioxidant properties, plays a crucial role in immunological defense, collagen synthesis, and wound healing [20]. A compromised concentration could impair therapeutic outcomes. particularly in pediatric patients with increased oxidative stress or limited endogenous reserves. Our findings reinforce the necessity for stringent quality control in compounding procedures and the importance of timely administration post-preparation. Moreover, the study has practical implications for home parenteral nutrition programs. In such contexts, deviations from cold-chain protocols may occur due to extended transportation times or inadequate caregiver education. Importantly, while the reduction in ascorbic acid content remained within the 10% pharmacopeial limit in most samples, cumulative degradation could become clinically relevant in long-term parenteral regimens, particularly when multiple vitamins are co-administered and stability profiles overlap. This further supports the need for individualized nutritional monitoring and more frequent assessment of micronutrient status in pediatric patients receiving home TPN [5]. Several measures can be recommended to mitigate the observed degradation. The incorporation of lipid emulsions not only meets caloric requirements but also offers additional photoprotection, as demonstrated in previous reports. Use of multilayered EVA bags with UV-blocking properties and minimizing headspace oxygen through nitrogen flushing or vacuum sealing can further enhance formulation stability [4]. Moreover, separating light-sensitive vitamins into co-infused secondary bags shortly before administration may present an additional solution, although this approach increases procedural complexity. From a regulatory and industrial perspective, these findings advocate for updates in shelf-life assignments and handling guidelines specific to pediatric TPN preparations. Regulatory agencies may consider mandating simulation studies that more accurately reflect real-world use, including storage in domestic refrigerators, transportation in non-controlled environments, and exposure during infusion. Harmonization of such protocols across institutions would ensure more consistent therapeutic outcomes. Future research should expand upon these findings by incorporating kinetic modeling of degradation under variable temperature profiles, assessing the interplay between pH shifts and oxidative degradation pathways, and evaluating stability in the presence of trace elements and transition metals that may catalyze vitamin oxidation. Additional attention should be given to the identification of degradation products and their potential toxicity, particularly since children represent a vulnerable population. Furthermore, given the emphasis on home-based medical care post-COVID-19 [22] and increasing decentralization of infusion therapy, the pharmaceutical industry should invest in innovation of container-closure systems that integrate indicators for temperature excursions, light exposure, and expiration tracking to empower end-users with actionable safety information. In conclusion, this study substantiates the partial degradation of ascorbic acid during typical storage and handling of pediatric TPN admixtures, particularly under room temperature exposure. The data underscore the importance of controlled storage conditions and informed handling to preserve vitamin potency. Although the observed reductions remained within pharmacopeial thresholds, the trend suggests a progressive decline that warrants attention in prolonged therapy scenarios. Our findings contribute to the growing body of evidence supporting stringent quality control in home parenteral nutrition and affirm the relevance of real-world conditions in stability evaluations. Ultimately, this research underscores the delicate balance between pharmaceutical formulation design, clinical utility, and patient safety. As the practice of parenteral nutrition continues to evolve, especially within home settings, a robust understanding of micronutrient stability will remain foundational to optimizing therapeutic outcomes for pediatric patients dependent on these life-sustaining therapies [7,23].

## Figures and Tables

**Table 1:** The composition of PN admixtures (unit—[g]).

TPN	*Glucose 50%*	*Aminoacids* *(Aminoven Infant)*	*Lipid Emulsion*	*Water for Injection*	*Glycophos^®^*	*10% NaCl*	*15% KCl*	*Peditrace^®^*	*20% MgSO_4_*	*Calcium* *Gluconicum*	*Soluvit^®^*	*Vitalipid^®^*	*Cernevit^®^*	*Supliven^®^*
101	58.7	58.7	18.3	91.6	1.6	5.3	4.4	0	1.8	7	1.8		0	0.7
102	66.3	66.3	26.3	15.6	2	4.5	1.8	2.9	0.4	5.5	2		0	0
103	59.1	53.2	32.5	88.7	0.7	4.4	3.3	0	1.8	4.4	1.5		0	0.4
104	69.5	65.5	40.1	45.4	0.8	10.7	9.6	0	1.1	5.3	0	0	0.7	1.3
105	88.4	68.7	33.7	36.5	0.6	4.2	3.5	2.1	1.1	7	2.8	1.4	0	0
106	58.4	44.9	24.7	95.4	0.8	4.9	6.7	0	2.8	9.7	0	0	0.6	1.1
107	75.1	44.7	26.8	89.4	0.4	2	1.6	2.7	0.5	3.2	1.8	1.8	0	0
108	88.9	86.9	46.8	10.7	0.9	2.9	3.3	0	1.3	7	0	0	0.7	0.5
109	81.2	43.8	10.3	72.2	1	4.1	2.1	2.3	0.3	5.9	2.3	2.6	0	0
110	94	82.9	49.8	2.8	0.8	3	3.3	0	1.1	7.6	1.4	2.8	0	0.6
111	60.2	40.1	10	103.6	1.3	2.7	2	2	0.3	5.7	2	3.3	0	0
112	68.4	63.5	28.2	63.5	2.1	3.7	6.5	1.4	1.4	8.5	1.4	1.4	0	0
113	56.9	59.4	33.4	78.6	1	6.3	2	0	1.7	7.8	1.2	1.2	0	0.5
114	89.9	62.1	42.8	40.7	1.1	1.5	1.5	3.2	0.9	4.3	2.1		0	0
115	114	67.3	47.8	4.3	0.4	4.3	0.9	3.3	0.7	4.8	2.2		0	0
116	70.8	43.6	28.6	89.9	0.5	3.3	1.6	2.2	0.5	4.1	2.2	2.7	0	0
117	95.6	82.2	41.1	10.1	0.3	3.4	1.7	2.5	1	8.7	1.7	1.7	0	0
118	56.5	64.5	35.6	72.6	0.1	5.4	3.4	0	2.4	7.5	1.3		0	0.7
119	58.7	58.7	18.3	91.6	1.6	5.3	4.4	0	1.8	7	1.8		0	0.7
120	103.4	66.3	26.3	15.6	2	4.5	1.8	2.9	0.4	5.5	2		0	0
121	59.1	53.2	32.5	88.7	0.7	4.4	3.3	0	1.8	4.4	1.5		0	0.4
122	69.5	65.5	40.1	45.4	0.8	10.7	9.6	0	1.1	5.3	0	0	0.7	1.3
123	88.4	68.7	33.7	36.5	0.6	4.2	3.5	2.1	1.1	7	1.4	2.8	0	0
124	58.4	44.9	24.7	95.4	0.8	4.9	6.7	0	2.8	9.7	0	0	0.6	1.1
125	75.1	44.7	26.8	89.4	0.4	2	1.6	2.7	0.5	3.2	1.8	1.8	0	0
126	88.9	86.9	46.8	10.7	0.9	2.9	3.3	0	1.3	7	0	0	0.7	0.5
127	81.2	43.8	10.3	72.2	1	4.1	2.1	2.3	0.3	5.9	2.3	2.6	0	0
128	94	82.9	49.8	2.8	0.8	3	3.3	0	1.1	7.6	1.4	2.8	0	0.6
129	60.2	40.1	10	103.6	1.3	2.7	2	2	0.3	5.7	2	3.3	0	0
130	68.4	63.5	28.2	63.5	2.1	3.7	6.5	1.4	1.4	8.5	1.4	1.4	0	0
131	56.9	59.4	33.4	78.6	1	6.3	2	0	1.7	7.8	1.2	1.2	0	0.5
132	89.9	62.1	42.8	40.7	1.1	1.5	1.5	3.2	0.9	4.3	2.1		0	0
133	114	67.3	47.8	4.3	0.4	4.3	0.9	3.3	0.7	4.8	2.2		0	0
134	70.8	43.6	28.6	89.9	0.5	3.3	1.6	2.2	0.5	4.1	2.2	2.7	0	0
135	95.6	82.2	41.1	10.1	0.3	3.4	1.7	2.5	1	8.7	1.7	1.7	0	0
136	56.5	64.5	35.6	72.6	0.1	5.4	3.4	0	2.4	7.5	1.3		0	0.7
137	61.2	35.8	14.9	121.2	1.2	3.6	1.2	0.7	0.4	5.4	1.5	3	0	0
138	61.2	35.8	7.8	113.4	1.2	3.6	1.2	0.7	0.4	5.4	1.5	3	0	0
139	90.1	68.4	34.2	34.2	2	6.5	5	1.6	0.8	6.5	0	0	0.8	0
140	79.2	69.7	57	14.3	1.7	13.1	3.2	1.6	0.6	7.9	1.6		0	0
141	69.1	68.6	38.4	54.9	0.5	2.3	3.1	1.1	1.1	9.2	0	0	0.5	0
142	44.9	23.5	6.8	141.1	1.1	4.2	2.3	1	1	2.6	1	1	0	0
143	81.4	48.1	15.7	53.6	1.7	5.7	2.8	1.4	0.2	8	1.8	1.8	0	0
144	100.5	68.6	43.4	13.7	1.8	3.4	2.1	1.8	1.8	8.2	2.3	2.3	0	0
145	60.4	40.9	5.6	130	2	0.9	3.3	1.3	0.4	1.5	1.9	1.9	0	0
146	102.8	58.6	22.6	26.7	2.9	2.5	2.1	1.4	0.7	7	2.1		0	0
147	43.2	37.8	4.8	131.9	1.2	4.6	1.3	0.9	0.7	2.2	1.2	1.2	0	0
148	87.2	89	51.3	5.4	1.4	2.3	2.5	1.8	0.9	6.3	1.8		0	0
149	55.1	42.5	22	110.2	1.6	1.3	1.6	1.9	0.3	7.2	3.1	3.1	0	0
150	70.9	59.1	25.9	34	1.2	7.1	3.7	1.5	0.9	5.9	1.5	1.5	0	0

**Table 2:** Lipid emulsion droplet size within TPN admixtures (DLS method) (D_x_ unit—[µm]).

Sample Number	SAMPLES “B”						SAMPLES “A”					
	t = 24 h			t = 24 h + 24 h			t = 15 days			t = 15 days + 24 h		
	Dx_(10)_	Dx_(50)_	Dx_(90)_	Dx_(10)_	Dx_(50)_	Dx_(90)_	Dx_(10)_	Dx_(50)_	Dx_(90)_	Dx_(10)_	Dx_(50)_	Dx_(90)_
101	0.067	0.198	0.474	0.084	0.222	0.474	0.071	0.205	0.480	0.072	0.206	0.485
102	0.158	0.290	0.462	0.157	0.283	0.449	0.174	0.295	0.447	0.171	0.294	0.452
103	0.066	0.207	0.538	0.113	0.252	0.467	0.066	0.196	0.462	0.090	0.233	0.488
104	0.144	0.277	0.458	0.142	0.275	0.458	0.109	0.250	0.477	0.101	0.244	0.480
105	0.100	0.241	0.476	0.130	0.276	0.491	0.091	0.233	0.487	0.097	0.246	0.502
106	0.065	0.192	0.453	0.077	0.208	0.448	0.077	0.217	0.501	0.072	0.211	0.500
107	0.161	0.290	0.459	0.157	0.285	0.454	0.158	0.286	0.453	0.156	0.285	0.455
108	0.145	0.278	0.461	0.128	0.263	0.458	0.116	0.256	0.470	0.125	0.268	0.486
109	0.161	0.288	0.454	0.164	0.287	0.448	0.179	0.298	0.445	0.173	0.295	0.448
110	0.138	0.277	0.474	0.105	0.244	0.460	0.145	0.285	0.485	0.083	0.223	0.482
111	0.161	0.289	0.457	0.167	0.291	0.451	0.177	0.296	0.447	0.171	0.293	0.449
112	0.109	0.248	0.462	0.121	0.264	0.480	0.092	0.234	0.485	0.100	0.248	0.501
113	0.058	0.180	0.447	0.061	0.190	0.476	0.084	0.227	0.499	0.084	0.229	0.502
114	0.137	0.274	0.469	0.111	0.258	0.497	0.078	0.215	0.480	0.102	0.254	0.516
115	0.157	0.287	0.458	0.138	0.275	0.465	0.170	0.294	0.454	0.162	0.289	0.455
116	0.158	0.288	0.459	0.113	0.251	0.460	0.172	0.296	0.455	0.149	0.279	0.453
117	0.142	0.276	0.459	0.116	0.258	0.476	0.106	0.246	0.466	0.122	0.265	0.479
118	0.074	0.209	0.473	0.117	0.258	0.476	0.058	0.180	0.450	0.087	0.226	0.470
119	0.096	0.227	0.436	0.103	0.230	0.424	0.089	0.216	0.426	0.092	0.219	0.425
120	0.133	0.255	0.418	0.152	0.269	0.421	0.151	0.268	0.420	0.150	0.267	0.419
121	0.094	0.225	0.437	0.101	0.227	0.422	0.072	0.194	0.437	0.080	0.207	0.424
122	0.130	0.247	0.404	0.128	0.245	0.403	0.090	0.212	0.406	0.106	0.229	0.411
123	0.116	0.245	0.436	0.128	0.252	0.426	0.113	0.237	0.420	0.114	0.239	0.422
124	0.102	0.229	0.423	0.106	0.228	0.408	0.074	0.199	0.417	0.069	0.192	0.417
125	0.145	0.262	0.420	0.137	0.253	0.407	0.121	0.241	0.408	0.115	0.234	0.403
126	0.137	0.253	0.406	0.137	0.252	0.403	0.127	0.247	0.414	0.128	0.247	0.411
127	0.159	0.280	0.439	0.162	0.279	0.430	0.127	0.287	0.433	0.167	0.282	0.429
128	0.120	0.245	0.426	0.134	0.254	0.416	0.129	0.248	0.411	0.133	0.254	0.418
129	0.153	0.274	0.435	0.156	0.276	0.430	0.167	0.283	0.432	0.156	0.274	0.425
130	0.121	0.246	0.428	0.116	0.241	0.422	0.095	0.222	0.424	0.085	0.211	0.421
131	0.101	0.227	0.425	0.115	0.239	0.419	0.089	0.216	0.424	0.082	0.207	0.417
132	0.132	0.254	0.421	0.129	0.249	0.416	0.128	0.250	0.420	0.109	0.233	0.414
133	0.122	0.244	0.413	0.136	0.253	0.406	0.150	0.262	0.405	0.138	0.251	0.401
134	0.142	0.259	0.412	0.126	0.244	0.404	0.105	0.227	0.411	0.112	0.234	0.412
135	0.145	0.261	0.414	0.135	0.255	0.417	0.103	0.229	0.421	0.119	0.245	0.431
136	0.119	0.242	0.422	0.116	0.240	0.421	0.085	0.214	0.432	0.090	0.219	0.437
137	0.189	0.304	0.446	0.169	0.289	0.442	0.162	0.283	0.440	0.171	0.289	0.441
138	0.171	0.293	0.448	0.160	0.283	0.445	0.152	0.279	0.448	0.132	0.263	0.447
139	0.137	0.274	0.468	0.135	0.272	0.465	0.126	0.268	0.475	0.120	0.262	0.477
140	0.180	0.295	0.438	0.153	0.276	0.436	0.176	0.290	0.432	0.171	0.287	0.433
141	0.092	0.226	0.453	0.118	0.256	0.461	0.103	0.246	0.481	0.108	0.252	0.486
142	0.165	0.286	0.444	0.140	0.266	0.439	0.150	0.276	0.445	0.144	0.273	0.448
143	0.171	0.291	0.444	0.151	0.278	0.447	0.164	0.285	0.444	0.165	0.288	0.447
144	0.172	0.299	0.459	0.142	0.275	0.458	0.112	0.251	0.466	0.132	0.274	0.478
145	0.167	0.297	0.468	0.156	0.290	0.469	0.144	0.276	0.455	0.157	0.287	0.457
146	0.174	0.296	0.451	0.159	0.287	0.455	0.161	0.284	0.446	0.168	0.290	0.448
147	0.148	0.281	0.464	0.165	0.284	0.436	0.144	0.269	0.438	0.169	0.290	0.445
148	0.143	0.278	0.464	0.093	0.231	0.460	0.096	0.235	0.464	0.129	0.272	0.485
149	0.079	0.209	0.443	0.070	0.195	0.437	0.062	0.184	0.447	0.066	0.190	0.449
150	0.163	0.286	0.446	0.161	0.285	0.446	0.132	0.262	0.444	0.158	0.283	0.447

**Table 3:** Droplet Size Distribution and Colloidal Stability Parameters of TPN Emulsions Measured by DLS.

Sample Number	SAMPLES “B”								SAMPLES “A”							
	t = 24 h				t = 24 h + 24 h				t = 15 days				t = 15 days + 24 h			
	Z-Average [µm]	PDI	ZETA Potential [mV]	Conductivity	Z-Average [µm]	PDI	ZETA Potential [mV]	Conductivity	Z-Average [µm]	PDI	ZETA Potential [mV]	Conductivity	Z-Average [µm]	PDI	ZETA Potential [mV]	Conductivity
101	240.900	0.208	−21.240	0.224	249.500	0.122	−26.360	0.097	233.750	0.114	−39.745	0.028	249.800	0.206	−31.410	0.032
102	245.650	0.135	−20.410	0.207	250.150	0.124	−23.670	0.087	234.000	0.123	−32.675	0.022	244.750	0.095	−32.055	0.022
103	241.300	0.132	−20.050	0.216	248.400	0.099	−25.130	0.090	239.400	0.119	−31.580	0.023	232.300	0.126	−31.760	0.024
104	239.900	0.110	−23.100	0.141	251.750	0.103	−23.360	0.114	238.050	0.142	−33.645	0.045	236.050	0.123	−34.305	0.047
105	245.750	0.125	−22.310	0.119	253.300	0.106	−22.650	0.106	240.650	0.119	−30.855	0.035	236.100	0.148	−33.190	0.029
106	241.400	0.103	−20.250	0.129	268.900	0.147	−22.970	0.116	234.900	0.103	−31.680	0.032	235.800	0.158	−28.335	0.035
107	240.800	0.132	−21.095	0.089	245.200	0.122	−23.630	0.098	238.900	0.128	−32.765	0.014	235.200	0.106	−33.985	0.014
108	233.600	0.092	−20.570	0.102	251.650	0.169	−22.560	0.107	235.900	0.071	−29.835	0.023	238.700	0.124	−32.840	0.024
109	252.500	0.086	−21.520	0.095	250.250	0.112	−21.490	0.106	248.400	0.152	−31.930	0.019	239.650	0.108	−31.015	0.019
110	249.400	0.123	−21.010	0.101	250.250	0.111	−21.320	0.108	240.800	0.110	−30.395	0.021	236.250	0.122	−33.110	0.024
111	244.050	0.109	−23.705	0.095	248.300	0.115	−23.680	0.103	247.400	0.136	−31.950	0.017	239.400	0.111	−32.780	0.017
112	240.850	0.118	−24.305	0.116	251.000	0.111	−24.100	0.115	237.600	0.123	−28.950	0.034	233.600	0.134	−31.670	0.034
113	253.600	0.158	−21.430	0.101	256.650	0.179	−22.805	0.112	236.900	0.123	−28.455	0.028	250.200	0.139	−31.385	0.029
114	248.700	0.124	−22.620	0.092	256.550	0.183	−23.220	0.101	235.350	0.098	−32.915	0.015	243.350	0.108	−33.360	0.013
115	250.400	0.116	−22.240	0.095	243.450	0.106	−23.240	0.103	234.450	0.077	−31.330	0.018	238.000	0.119	−32.825	0.175
116	246.900	0.139	−22.360	0.092	247.450	0.143	−25.410	0.102	228.450	0.114	−32.155	0.016	241.550	0.119	−31.695	0.016
117	230.200	0.130	−17.250	0.188	236.300	0.142	−21.660	0.083	238.250	0.091	−29.145	0.021	238.950	0.127	−29.825	0.023
118	251.950	0.181	−19.220	0.206	233.400	0.107	−22.450	0.095	237.350	0.113	−30.025	0.029	247.950	0.105	−30.820	0.028
119	221.150	0.991	−16.830	0.206	215.600	0.074	−20.680	0.096	226.400	0.101	−26.960	0.033	225.400	0.092	−30.195	0.032
120	225.400	1.000	−17.860	0.198	222.450	0.035	−21.460	0.099	226.100	0.017	−31.005	0.023	228.500	0.048	−28.755	0.026
121	211.650	0.777	−16.145	0.203	208.300	0.065	−18.880	0.108	223.600	0.079	−26.930	0.027	227.350	0.058	−30.345	0.027
122	220.400	0.133	−16.720	0.221	209.150	0.044	−19.400	0.106	218.700	0.062	−25.325	0.048	221.700	0.047	−26.275	0.056
123	221.750	0.121	−12.730	0.199	210.800	0.077	−21.840	0.079	221.200	0.097	−27.305	0.026	223.950	0.057	−27.230	0.031
124	215.950	0.116	−18.180	0.215	215.650	0.062	−30.650	0.091	220.000	0.074	−23.120	0.034	229.550	0.057	−23.985	0.037
125	214.700	0.078	−15.120	0.191	209.700	0.071	−20.740	0.069	225.550	0.119	−28.045	0.016	234.050	0.150	−30.340	0.017
126	217.450	0.085	−15.170	0.201	212.850	0.039	−20.800	0.078	218.300	0.077	−27.840	0.027	221.600	0.065	−29.290	0.029
127	227.050	0.049	−16.980	0.198	222.550	0.094	−22.180	0.075	234.300	0.094	−29.565	0.021	232.900	0.105	−30.105	0.022
128	221.050	0.085	−15.110	0.202	219.750	0.118	−22.980	0.081	224.250	0.074	−27.110	0.024	217.550	0.087	−28.450	0.026
129	226.100	0.144	−17.680	0.197	225.400	0.125	−23.440	0.074	235.650	0.115	−29.805	0.019	233.250	0.079	−28.940	0.020
130	219.250	0.063	−17.220	0.210	219.200	0.143	−16.790	0.155	226.050	0.073	−25.740	0.037	224.450	0.073	−25.765	0.040
131	216.550	0.077	−13.930	0.205	212.850	0.055	−14.490	0.146	226.450	0.089	−27.905	0.026	221.000	0.096	−29.345	0.029
132	235.350	0.183	−16.300	0.195	221.150	0.111	−22.680	0.085	227.150	0.035	−28.300	0.016	224.550	0.063	−28.940	0.019
133	210.800	0.080	−14.140	0.198	209.050	0.050	−16.070	0.136	219.750	0.073	−28.665	0.019	225.550	0.077	−26.145	0.020
134	217.800	0.144	−15.370	0.173	208.500	0.057	−15.485	0.113	212.000	0.087	−29.350	0.021	220.200	0.073	−29.255	0.020
135	209.550	0.098	−15.380	0.177	208.650	0.067	−15.390	0.116	220.500	0.085	−28.450	0.022	219.100	0.079	−30.640	0.022
136	212.700	0.030	−12.940	0.184	214.800	0.074	−17.650	0.110	216.400	0.071	−28.370	0.035	219.550	0.061	−32.450	0.003
137	228.200	0.075	−17.520	−0.167	232.800	0.133	−21.220	0.099	239.950	0.131	−33.300	0.018	238.000	0.138	−31.165	0.020
138	228.050	0.115	−17.220	0.171	229.000	0.090	−20.740	0.100	252.500	0.176	−31.740	0.018	243.700	0.124	−31.045	0.019
139	223.300	0.110	−17.015	0.186	224.150	0.079	−20.050	0.104	237.250	0.138	−33.835	0.035	236.800	0.131	−31.725	0.032
140	228.550	0.097	−16.105	0.188	234.150	0.090	−20.015	0.107	235.250	0.113	−32.850	0.039	236.000	0.106	−35.295	0.045
141	227.050	0.103	−15.250	0.174	227.300	0.121	−21.095	0.088	247.750	0.109	−29.510	0.023	241.150	0.126	−32.190	0.028
142	229.500	0.138	−15.740	0.170	229.350	0.090	−18.900	0.087	249.600	0.141	−31.835	0.020	239.700	0.102	−33.690	0.021
143	231.700	0.128	−17.655	0.174	227.800	0.134	−20.665	0.092	249.750	0.130	−34.000	0.026	237.850	0.074	−31.815	0.027
144	229.600	0.086	−15.925	0.170	227.800	0.102	−20.845	0.091	237.900	0.125	−29.580	0.024	239.100	0.119	−27.390	0.022
145	225.700	0.137	−16.890	0.169	240.750	0.161	−16.465	0.154	233.750	0.131	−35.915	0.017	136.750	0.219	−36.660	0.021
146	229.950	0.123	−16.465	0.177	231.400	0.131	−15.995	0.160	232.150	0.099	−31.905	0.022	239.800	0.124	−29.850	0.032
147	228.300	0.104	−16.310	0.170	233.500	0.113	−16.455	0.159	255.950	0.184	−34.925	0.020	236.400	0.078	−35.115	0.043
148	229.200	0.097	−16.040	0.177	233.000	0.116	−15.420	0.160	234.300	0.141	−31.455	0.022	249.850	0.165	−29.940	0.021
149	218.250	0.131	−16.030	0.170	229.650	0.122	−15.915	0.156	240.700	0.108	−36.150	0.015	253.200	0.189	−31.695	0.023
150	230.100	0.099	−16.555	0.184	234.450	0.078	−17.205	0.171	240.750	0.130	−31.875	0.030	239.350	0.115	−32.995	0.031

**Table 4:** The pH values of all admixtures.

Sample Number	SAMPLES “B”		SAMPLES “A”	
	t = 24 h	t = 24 h + 24 h	t = 15 days	t = 15 days + 24 h
101	6.06	5.97	5.87	6.04
102	5.98	6.00	5.98	5.96
103	6.07	6.01	5.94	5.90
104	6.13	5.95	6.05	5.94
105	6.14	5.89	5.95	5.92
106	5.87	5.95	6.02	6.14
107	5.92	5.89	5.97	6.01
108	6.13	6.03	5.91	6.08
109	5.98	5.96	6.06	6.02
110	6.08	5.90	6.07	6.11
111	5.86	5.95	5.92	5.89
112	6.00	5.97	6.13	6.03
113	5.89	5.87	6.00	6.05
114	6.07	6.01	6.10	5.94
115	6.13	5.95	5.95	6.05
116	5.89	5.94	6.04	5.98
117	6.15	6.07	6.03	6.16
118	5.93	6.15	5.98	5.96
119	6.08	6.11	5.86	6.08
120	6.13	6.07	5.97	6.01
121	5.89	6.12	5.91	6.08
122	5.95	5.89	5.92	6.09
123	6.14	5.96	6.11	6.08
124	5.87	5.95	5.92	6.11
125	6.00	6.02	6.14	6.15
126	6.07	6.03	6.16	5.87
127	5.97	6.12	5.96	6.14
128	6.12	5.89	6.12	5.91
129	5.89	5.95	5.89	5.92
130	6.02	6.06	6.02	6.02
131	6.11	6.07	6.11	5.95
132	5.89	6.09	5.90	6.00
133	6.03	6.00	5.88	6.10
134	5.94	5.90	5.94	5.95
135	6.12	6.13	6.11	6.04
136	6.01	6.00	6.05	5.99
137	5.96	6.10	5.94	5.90
138	6.08	5.95	6.05	5.94
139	5.88	6.04	5.98	6.05
140	5.89	6.12	6.02	6.09
141	6.17	5.89	6.12	6.06
142	5.89	5.89	6.08	5.95
143	5.88	5.96	6.12	5.94
144	6.06	5.97	6.04	6.03
145	6.00	6.01	5.96	6.14
146	5.89	5.95	5.99	5.87
147	6.17	5.89	6.12	6.05
148	5.89	5.89	6.00	5.88
149	5.99	6.01	5.92	5.90
150	5.91	6.02	6.12	5.86

**Table 5:** Quantitative determination of ascorbic acid using a validated HPLC method (unit—[mg/mL]), and vitamin content [% of initial concentration (T = 0)].

Sample Number	T = 0	T = 24 h	T = 24 + 24 h	T = 15 days	T = 15 days + 24 h
101	0.0720	0.0691 (96.00%)	0.0691 (96.00%)	0.0677 (94.12%)	0.0670 (93.18%)
102	0.0850	0.0816 (98.17%)	0.0816 (94.17%)	0.0800 (93.50%)	0.0792 (91.17%)
103	0.0600	0.0589 (95.29%)	0.0565 (93.57%)	0.0561 (91.00%)	0.0547 (94.29%)
104	0.0700	0.0667 (97.68%)	0.0655 (99.64%)	0.0637 (98.04%)	0.0660 (93.21%)
105	0.0560	0.0547 (98.00%)	0.0558 (96.17%)	0.0549 (96.67%)	0.0522 (95.83%)
106	0.0600	0.0588 (97.22%)	0.0577 (95.97%)	0.0580 (97.50%)	0.0575 (93.19%)
107	0.0720	0.0700 (99.57%)	0.0691 (98.57%)	0.0702 (96.00%)	0.0671 (92.43%)
108	0.0700	0.0697 (95.98%)	0.0690 (95.98%)	0.0672 (94.13%)	0.0647 (93.15%)
109	0.0920	0.0883 (96.00%)	0.0883 (96.00%)	0.0866 (94.00%)	0.0857 (93.17%)
110	0.0600	0.0576 (96.00%)	0.0576 (96.00%)	0.0564 (94.13%)	0.0559 (93.13%)
111	0.0800	0.0768 (96.07%)	0.0768 (96.07%)	0.0753 (94.11%)	0.0745 (93.04%)
112	0.0560	0.0538 (96.04%)	0.0538 (96.04%)	0.0527 (94.17%)	0.0521 (93.13%)
113	0.0480	0.0461 (96.07%)	0.0461 (96.07%)	0.0452 (94.05%)	0.0447 (93.10%)
114	0.0840	0.0807 (98.63%)	0.0807 (95.91%)	0.0790 (94.89%)	0.0782 (97.73%)
115	0.0880	0.0868 (95.57%)	0.0844 (96.48%)	0.0835 (96.02%)	0.0860 (99.20%)
116	0.0880	0.0841 (96.91%)	0.0849 (95.15%)	0.0845 (97.94%)	0.0873 (94.41%)
117	0.0680	0.0659 (97.88%)	0.0647 (93.65%)	0.0666 (97.69%)	0.0642 (95.96%)
118	0.0520	0.0509 (98.61%)	0.0487 (97.50%)	0.0508 (96.81%)	0.0499 (98.06%)
119	0.0720	0.0710 (99.00%)	0.0702 (98.50%)	0.0697 (98.38%)	0.0706 (93.88%)
120	0.0800	0.0792 (94.67%)	0.0788 (94.50%)	0.0787 (94.00%)	0.0751 (96.50%)
121	0.0600	0.0568 (98.14%)	0.0567 (96.43%)	0.0564 (95.00%)	0.0579 (90.29%)
122	0.0700	0.0687 (96.07%)	0.0675 (96.07%)	0.0665 (94.11%)	0.0632 (93.04%)
123	0.0560	0.0538 (96.00%)	0.0538 (96.00%)	0.0527 (94.00%)	0.0521 (93.17%)
124	0.0600	0.0576 (95.97%)	0.0576 (95.97%)	0.0564 (94.03%)	0.0559 (93.06%)
125	0.0720	0.0691 (96.00%)	0.0691 (96.00%)	0.0677 (94.14%)	0.0670 (93.14%)
126	0.0700	0.0672 (95.98%)	0.0672 (95.98%)	0.0659 (94.13%)	0.0652 (93.15%)
127	0.0920	0.0883 (96.00%)	0.0883 (96.00%)	0.0866 (94.00%)	0.0857 (93.17%)
128	0.0600	0.0576 (96.00%)	0.0576 (96.00%)	0.0564 (94.13%)	0.0559 (93.13%)
129	0.0800	0.0768 (96.07%)	0.0768 (96.07%)	0.0753 (94.11%)	0.0745 (93.04%)
130	0.0560	0.0538 (96.04%)	0.0538 (96.04%)	0.0527 (94.17%)	0.0521 (93.13%)
131	0.0480	0.0461 (96.67%)	0.0461 (96.19%)	0.0452 (96.67%)	0.0447 (95.12%)
132	0.0840	0.0812 (98.75%)	0.0808 (98.75%)	0.0812 (97.16%)	0.0799 (96.48%)
133	0.0880	0.0869 (93.52%)	0.0869 (95.91%)	0.0855 (91.70%)	0.0849 (91.36%)
134	0.0880	0.0823 (95.29%)	0.0844 (93.09%)	0.0807 (91.91%)	0.0804 (92.50%)
135	0.0680	0.0648 (98.46%)	0.0633 (96.54%)	0.0625 (96.73%)	0.0629 (96.92%)
136	0.0520	0.0512 (96.00%)	0.0502 (96.00%)	0.0503 (94.00%)	0.0504 (93.17%)
137	0.0600	0.0576 (97.50%)	0.0576 (93.33%)	0.0564 (92.00%)	0.0559 (90.17%)
138	0.0600	0.0585 (99.13%)	0.0560 (93.13%)	0.0552 (95.00%)	0.0541 (97.75%)
139	0.0800	0.0793 (97.34%)	0.0745 (99.69%)	0.0760 (95.94%)	0.0782 (92.97%)
140	0.0640	0.0623 (99.80%)	0.0638 (97.20%)	0.0614 (98.80%)	0.0595 (99.20%)
141	0.0500	0.0499 (96.25%)	0.0486 (94.75%)	0.0494 (93.25%)	0.0496 (91.50%)
142	0.0400	0.0385 (94.44%)	0.0379 (95.83%)	0.0373 (92.78%)	0.0366 (94.03%)
143	0.0720	0.0680 (94.89%)	0.0690 (97.07%)	0.0668 (91.74%)	0.0677 (90.65%)
144	0.0920	0.0873 (96.32%)	0.0893 (95.79%)	0.0844 (92.24%)	0.0834 (90.92%)
145	0.0760	0.0732 (95.12%)	0.0728 (96.19%)	0.0701 (96.67%)	0.0691 (95.12%)
146	0.0840	0.0799 (92.71%)	0.0808 (98.13%)	0.0812 (94.38%)	0.0799 (95.42%)
147	0.0480	0.0445 (95.56%)	0.0471 (94.17%)	0.0453 (93.33%)	0.0458 (91.11%)
148	0.0720	0.0688 (95.81%)	0.0678 (93.79%)	0.0672 (93.47%)	0.0656 (92.34%)
149	0.1240	0.1188 (95.00%)	0.1163 (96.83%)	0.1159 (93.67%)	0.1145 (92.17%)
150	0.0600	0.0570 (95.00%)	0.0581 (96.83%)	0.0562 (93.67%)	0.0553 (92.17%)

## Data Availability

Data available on request.
